# COVID-19 Vaccine Hesitancy and Trust in Government in Nigeria

**DOI:** 10.3390/vaccines10071008

**Published:** 2022-06-23

**Authors:** Ryoko Sato

**Affiliations:** Harvard T.H. Chan School of Public Health, Center for Health Decision Science, Department of Global Health and Population, 90 Smith Street, Boston, MA 02120, USA; rsato@hsph.harvard.edu or ryokos1226@gmail.com; Tel.: +1-202-290-5064

**Keywords:** vaccine hesitancy, COVID-19 vaccine, trust, government, Nigeria

## Abstract

Introduction: COVID-19 has been impacting our lives globally, including in Nigeria. While the COVID-19 vaccine is available free of charge, vaccination coverage remains low. This study evaluates the relationship between trust in government and COVID-19 vaccine hesitancy. Methods: We used an Afrobarometer survey for data on trust in government and the COVID-19 National Longitudinal Phone Survey (NLPS) for data on COVID-19 vaccine hesitancy, merged by strata (states and urban/rural). The simple correlation was evaluated using Ordinary Least Squares (OLS) regression. Results: Distrust in government was strongly associated with COVID-19 vaccine hesitancy as well as with perceptions that the vaccine was not safe, and concerns about side effects were given as reasons for vaccine refusal. Discussion/Conclusion: Distrust of government is an important predictor of vaccine hesitancy in Nigeria. This result is consistent with findings in the literature, especially in developed countries. Vaccine refusers, who distrust the government, refuse vaccines because they think that vaccines do them harm. Policy makers should be cautious when it comes to strategizing for COVID-19 vaccine distribution, especially in places where trust in government is weak.

## 1. Introduction

COVID-19 has been impacting our lives globally. By the beginning of 2022, there were over 430 million cases of COVID-19 and about 6 million deaths due to COVID-19 worldwide [1]. In Nigeria, which has the largest population in sub-Saharan Africa, COVID-19 cases reached more than 250,000, with over 3000 deaths [1].

Thanks to technological advancement and intensive investment, COVID-19 vaccines became available worldwide rapidly [2]. In Nigeria, COVID-19 vaccines became available early in 2021 at no cost to recipients [3]. However, the number of vaccines administered has been limited. So far, about 25 million doses of vaccines have been administered, which would cover around 5% of the population with two doses [1]. This low vaccination coverage might be due to various barriers, one of which is vaccine hesitancy, as some previous studies have pointed out within the Nigerian context [4,5].

Nigeria has a history of vaccine hesitancy [6,7,8,9,10]. One of the most well-known incidences of vaccine hesitancy in Nigeria was the boycott of the polio vaccination campaign that occurred in northern Nigeria in 2003 [11,12,13]. The boycott lasted 16 months and this movement resulted in the spread of polio infections within the country, as well as across other neighboring countries [14]. Nigeria was one of a few countries that had not eradicated wild polio in 2020 [15]. One of the important reasons for this boycott is believed to be related to public trust and especially distrust of the federal government, which advocated the polio eradication campaign.

In the current COVID-19 pandemic, COVID-19 vaccination is strongly recommended by public entities in almost all countries. In Nigeria, the (lack of) public trust once again might play an important role in the (un)successful vaccination campaign against COVID-19, especially because Nigeria struggles with government trust [16,17,18]. This study evaluates the relationship between trust in government and COVID-19 vaccine hesitancy.

## 2. Methods

### 2.1. Data Used for the Analysis

To evaluate the correlation between trust in government and COVID-19 vaccine hesitancy, we used two datasets. One was the Afrobarometer survey (Round 7) conducted in various African countries, including Nigeria. The Afrobarometer survey intended to capture national public attitudes towards democracy, governance, and society. It asked various questions relating to public perceptions, including trust in government and other institutions. In this study, we focused exclusively on questions related to trust. In the survey, the extent to which an individual trusted various institutions was measured with the following question: “How much do you trust each of the following, or haven’t you heard enough about them to say?”. The categories of entity this study used to measure trust included the president, the parliament, the ruling political party, traditional leaders, and religious leaders. Responses could be “Not at all”, “Just a little”, “Somewhat”, “A lot”, and “Don’t know”. In this analysis, we focused on “Not at all”, as this response can be interpreted as expressing strong distrust in the entity in question, and “A lot”, as this response reflects a strong trust in the entity.

In Nigeria, the Afrobarometer survey was conducted among nationally representative samples in 2017, and this round of the survey was the most recent one that was available at the time of the data analysis. The current Nigerian president has been in office since 2015; thus, the responses about trust in the president, the parliament, and the ruling political party should still be valid when examining their relationship to COVID-19 vaccine hesitancy.

Another dataset we used was the COVID-19 National Longitudinal Phone Survey (NLPS) conducted among nationally representative samples in Nigeria. The sample was also representative of six geopolitical zones. This was a longitudinal study that followed the same respondents over time. The total of 1950 households were followed-up from Round 1 to Round 12. Each cycle from Round 1 to Round 12 was conducted every month. In this analysis, we used the data from Round 6, which was conducted in 2020 October, because during the Round 6 survey the module on perceptions and attitudes toward COVID-19 vaccine was first introduced. In particular, NLPS Round 6 included the following survey question: “Would you agree to being vaccinated against COVID-19 if a vaccine was available right now at no cost to you?” We used the answer to this question to measure hesitancy in relation to COVID-19 vaccination. The answer could be “Yes”, “No”, or “Not sure”. Both answers “No” and “Not sure” were examined separately. At the time of the survey, the COVID-19 vaccine was not available, and thus the question regarding actual vaccine uptake was not available.

If a respondent answered “No” to this question, they were then asked, “Why would not you agree to be vaccinated?”. They could answer either “Yes” or “No” for the following potential reasons: “Vaccine does not work”, “Vaccine is not safe”, “Fear of side effects”, “Low risk of contracting COVID-19”.

### 2.2. Statistical Analysis

To evaluate the correlation between trust in government and COVID-19 vaccine hesitancy, we merged the information from the Afrobarometer survey and the information from the NLPS based on the geographical regions in which respondents were interviewed.

In Nigeria, there are 36 states and 1 federal capital territory. Most states can be divided into urban and rural areas, while some states only have either urban or rural areas. In total, we had 71 strata (combinations of states and urban/rural areas).

For each stratum, we merged the average level of trust (various variables) among respondents who were surveyed in the stratum and the average level of vaccine hesitancy variables among respondents in the same stratum. While these two surveys were conducted among different populations, thus it is infeasible to evaluate the correlation at an individual level, it is still possible to analyze the correlations using the average level of vaccine hesitancy and trust in each stratum, as both surveys were based on nationally representative samples. Such merging methods are commonly described elsewhere [19,20]. Then, we evaluated the correlations between them using simple Ordinary Least Squares (OLS) regression.

## 3. Results

Table 1, Panel A presents the correlations between distrust in government and COVID-19 vaccine hesitancy. If a respondent had no trust at all in the president, parliament, the ruling party, or traditional leaders, she had a higher likelihood of being against vaccination. Distrust in religious leaders was not correlated with vaccine hesitancy. Similarly, if a respondent had no trust at all in the president, parliament, or the ruling party, she had a higher likelihood of being unsure whether she would receive a COVID-19 vaccine. Distrust in the government was more strongly correlated with vaccine refusal (being against vaccination) than with indecision (being unsure about vaccination).

Table 1, Panel B presents the correlations between trust in government and COVID-19 vaccine hesitancy. The results in Panel B are consistent with the results in Panel A. If a respondent had a strong trust in the president, parliament, the ruling party, or traditional leaders, she had a lower likelihood of being against vaccination. A strong trust in religious leaders was not correlated with vaccine hesitancy. Similarly, if a respondent had a strong trust in the president, parliament, the ruling party, or traditional leaders, she had a lower likelihood of being unsure whether she would receive a COVID-19 vaccine. Trust in the government was more strongly and negatively correlated with vaccine refusal (being against vaccination) than with indecision (being unsure about vaccination).

The analyses so far have been conducted for both urban and rural areas. When the urban and rural samples were analyzed separately, we found that the negative correlation between trust and vaccine hesitancy was predominantly derived from the rural samples rather than the urban samples. Among urban samples, we found no or only a very weak correlation.

Table 2, Panel A presents the correlations between distrust in government and reasons for vaccine hesitancy among those who stated that they would refuse a COVID-19 vaccine when one became available for free. Those with a strong distrust in government, especially in the president, were less likely to be against vaccination due to a belief that the COVID-19 vaccine would not work (Column 1) or due to a belief that there was only a low risk of disease contraction, although the results were not statistically significant (Columns 16 to 18). Rather, those with strong distrust of the government, especially parliament and the ruling party, were more likely to be against vaccination due to a belief that the COVID-19 vaccine was not safe (Columns 7 and 8) or due to a concern about side effects, although the results were again not statistically significant (Columns 11 to 13).

Similar to the sub-group analysis conducted for Table 1, when the urban and rural samples were analyzed separately we found consistent and stronger results, as can be seen in Table 2, among rural samples as compared with urban samples.

Table 2, Panel B presents the correlations between trust in government and the reasons for vaccine hesitancy among those who refused to receive a COVID-19 vaccine when one became available for free. Those with a strong trust in the government, especially in the president and the ruling party, were more likely to be against vaccination due to a belief that the COVID-19 vaccine would not work (Column 1) or due to a belief that there was only a low risk of disease contraction, although the results were not statistically significant (Column 17). On the other hand, those with a strong trust in the government, especially in the ruling party, were less likely to be against vaccination due to a belief that the COVID-19 vaccine was not safe (Column 8), or due to a concern about side effects (Column 13).

## 4. Discussion

This study has evaluated correlations between the COVID-19 vaccine hesitancy and trust in government in Nigeria. We used two separate household surveys that were publicly available and merged them based on respondents’ geographical locations.

We found that the more that people distrusted government, the more likely they were to be against receiving the COVID-19 vaccine (vaccine hesitancy). Distrust of government was also positively correlated with the likelihood that people were unsure about receiving the COVID-19 vaccine (vaccine indecisiveness), but the correlation was weaker as compared to the correlation between distrust in government and vaccine hesitancy.

Consistently, if an individual had strong trust in the government, they were less likely to be against receiving a COVID-19 vaccine and to be unsure about it. These results indicate that distrust in government is an important predictor of vaccine hesitancy.

This result is consistent with some findings in the literature, especially in developed countries [21,22,23,24], with some exceptions [25,26]. There are similar findings in more global contexts as well [27].

There is extremely limited evidence on the relationship between trust in government and COVID-19 vaccine hesitancy in Africa in the literature [28]. This study contributes to the literature by providing additional evidence of the strong link between public distrust and COVID-19 vaccine hesitancy in the African context. Nigeria, in particular, is of great importance because it has the largest population in Africa and it has been struggling with low vaccination coverage in the context of more established child vaccination programs [29]. Understanding the underlying cause of vaccine hesitancy is of particular importance in Nigeria in order to mitigate societal losses due to non-vaccination.

This study further investigated the link between distrust in government and reasons for vaccine hesitancy among those who refused to receive the COVID-19 vaccine. This area of study is extremely limited, yet it is of critical importance to understand why vaccine hesitaters refuse the vaccine in the African context.

Among people who expressed COVID-19 vaccine hesitancy, the study found that distrust in government was significantly and positively correlated with the likelihood of perceiving the COVID-19 vaccine to be not safe and not believing that the vaccine works. On the other hand, distrust in government was not correlated with other reasons for vaccine hesitancy, such as fear of side effects of a COVID-19 vaccine and low perceptions of the risk of contracting COVID-19.

On the other hand, if people who refused to receive a COVID-19 vaccine had strong trust in government, they were more likely to believe that the vaccine does not work and that they had a low risk of contracting COVID-19. On the other hand, they were more likely to believe that the vaccine was safe, while being less likely to have fears of side effects.

These results on the relationship between trust in government and reasons for vaccine hesitancy reveal that hesitant people who distrust the government refuse vaccines because they think that vaccines are bad for them; they tend to believe that vaccines are not safe and they are concerned about side effects, but not because they think that vaccines do not work or because of low risk perceptions. In other words, distrust of government is associated with the perception that the COVID-19 vaccine is harmful to them. This result might indicate that these vaccine hesitaters perceive the government to be offering COVID-19 vaccines to harm the population.

Finally, the main results regarding the correlation between trust and vaccine hesitancy were found to be relevant in rural settings but not in urban settings. This is an interesting finding but identifying the reasons for the differential results was beyond the scope of the study. A future study should investigate the potential reasons for this difference.

### Limitations

This study has various limitations. First, trust in government is endogenous, and thus this study does not detect the causal relationship between trust in government and COVID-19 vaccine hesitancy. Second, because we merged two different datasets by strata (state and urban/rural areas), the number of observations is limited. Furthermore, trust variables are matched with vaccine hesitancy variables for each stratum, but not at an individual level; thus, this study only analyzed average relationships by strata. Since the surveys were based on nationally representative samples, they were not representative at each stratum; this sampling frame implies that the averages of independent and dependent variables in each survey might be biased. Thus, the interpretation of results needs caution. Finally, it is worth noting that the COVID-19 pandemic was perceived to be not as severe in many African countries, including Nigeria, as in other countries. The low prevalence of the disease could have influenced the level of vaccine hesitancy and thus the correlation between trust and vaccine hesitancy. The prevalence of COVID-19 could have been impacted by various factors, including climate [30,31,32]. However, in this study, in which we focused only on one country, it was beyond the scope of the study to investigate the impact of other factors on the correlation between trust and vaccine hesitancy, despite its importance.

## 5. Conclusions

The study confirmed the important relationship between trust in government and COVID-19 vaccine hesitancy in Nigeria. In particular, we found that trust in government was correlated with lower levels of COVID-19 vaccine hesitancy, especially in rural areas. Those who distrusted the government were more likely to believe that COVID-19 vaccines were harmful to them. Policy makers should be cautious when it comes to strategizing for COVID-19 vaccine distribution, especially in places where trust in government is weak. For example, countering mis/disinformation about COVID-19 vaccines might be crucial in efforts to improve COVID-19 vaccine uptake by mitigating vaccine hesitancy.

## Figures and Tables

**Table 1:** Trust and vaccine hesitancy.

	Against Vaccination					Not Sure about Vaccination				
	(1)	(2)	(3)	(4)	(5)	(6)	(7)	(8)	(9)	(10)
*Panel A: No trust at all in*										
President	0.174 ***					0.104 ***				
	(0.058)					(0.028)				
Parliament		0.150 **					0.063 *			
		(0.067)					(0.033)			
Ruling party			0.174 ***					0.058 *		
			(0.059)					(0.030)		
Traditional leaders				0.183 **					0.037	
				(0.088)					(0.045)	
Religious leaders					0.210					0.021
					(0.129)					(0.065)
_cons	0.069 ***	0.059 *	0.059 **	0.086 ***	0.097 ***	−0.006	−0.001	0.005	0.018	0.023 **
	(0.022)	(0.031)	(0.025)	(0.022)	(0.021)	(0.011)	(0.016)	(0.013)	(0.011)	(0.011)
N	71	71	71	71	71	71	71	71	71	71
r2	0.116	0.069	0.112	0.059	0.037	0.167	0.050	0.050	0.010	0.002
	Against vaccination					Not sure about vaccination				
	(1)	(2)	(3)	(4)	(5)	(6)	(7)	(8)	(9)	(10)
*Panel B: Strong trust in*										
President	−0.148 **					−0.049 *				
	(0.057)					(0.029)				
Parliament		−0.322 **					−0.133*			
		(0.155)					(0.078)			
Ruling party			−0.339 ***					−0.142 ***		
			(0.105)					(0.054)		
Traditional leaders				−0.243 ***					−0.109 **	
				(0.090)					(0.045)	
Religious leaders					−0.095					−0.045
					(0.069)					(0.034)
_cons	0.161 ***	0.147 ***	0.169 ***	0.163 ***	0.156 ***	0.038 ***	0.036 ***	0.045 ***	0.044 ***	0.042 ***
	(0.020)	(0.019)	(0.020)	(0.021)	(0.028)	(0.010)	(0.009)	(0.010)	(0.010)	(0.014)
N	71	71	71	71	71	71	71	71	71	71
r2	0.089	0.058	0.131	0.095	0.027	0.039	0.041	0.092	0.078	0.025

Notes: * Significant at 10%. ** Significant at 5%. *** Significant at 1%.

**Table 2:** Trust and reasons for vaccine hesitancy.

	Vaccine Does Not Work					Vaccine Is Not Safe					Fear of Side Effects					Low Risk of Contracting COVID-19				
	(1)	(2)	(3)	(4)	(5)	(6)	(7)	(8)	(9)	(10)	(11)	(12)	(13)	(14)	(15)	(16)	(17)	(18)	(19)	(20)
*Panel A: No trust at all in:*																				
President	−0.250 *					0.191					0.113					−0.089				
	(0.128)					(0.173)					(0.139)					(0.184)				
Parliament		−0.193					0.334 *					0.110					−0.188			
		(0.153)					(0.199)					(0.162)					(0.214)			
Ruling party			−0.217					0.347 *					0.211					−0.271		
			(0.135)					(0.177)					(0.143)					(0.190)		
Traditional leaders				−0.279					0.303					0.022					−0.165	
				(0.192)					(0.255)					(0.206)					(0.272)	
Religious leaders					−0.545 *					0.253					−0.033					0.227
					(0.279)					(0.379)					(0.304)					(0.402)
N	57	57	57	57	57	57	57	57	57	57	57	57	57	57	57	57	57	57	57	57
r2	0.065	0.028	0.045	0.037	0.065	0.022	0.049	0.065	0.025	0.008	0.012	0.008	0.038	0.000	0.000	0.004	0.014	0.036	0.007	0.006
	Vaccine does not work					Vaccine is not safe					Fear of side effects					Low risk of contracting COVID-19				
	(1)	(2)	(3)	(4)	(5)	(6)	(7)	(8)	(9)	(10)	(11)	(12)	(13)	(14)	(15)	(16)	(17)	(18)	(19)	(20)
*Panel B: Strong trust in:*																				
President	0.213 *					−0.277					−0.276 **					0.276				
	(0.127)					(0.168)					(0.132)					(0.178)				
Parliament		0.077					−0.672					−0.122					0.956 *			
		(0.365)					(0.473)					(0.384)					(0.493)			
Ruling party			0.574 **					−0.585 *					−0.480 *					0.465		
			(0.230)					(0.310)					(0.247)					(0.333)		
Traditional leaders				0.478 **					−0.120					−0.353 *					0.020	
				(0.189)					(0.263)					(0.205)					(0.279)	
Religious leaders					0.395 **					−0.058					−0.288 *					0.135
					(0.152)					(0.212)					(0.165)					(0.224)
N	57	57	57	57	57	57	57	57	57	57	57	57	57	57	57	57	57	57	57	57
r2	0.049	0.001	0.101	0.104	0.110	0.047	0.035	0.061	0.004	0.001	0.074	0.002	0.064	0.051	0.052	0.042	0.064	0.034	0.000	0.007

* Significant at 10%. ** Significant at 5%. *** Significant at 1%.

## Data Availability

Publicly available datasets were analyzed in this study. This data can be found here: https://www.afrobarometer.org/data/ (accessed on 14 May 2022); https://microdata.worldbank.org/index.php/catalog/3712 (accessed on 14 May 2022).
